# The effects of tarantula cubensis extract on renal ischemia reperfusion injury in rats

**DOI:** 10.55730/1300-0144.5606

**Published:** 2023-10-09

**Authors:** Saime ÖZBEK ŞEBİN, TUNCER NACAR, Ayhan TANYELİ, Ersen ERASLAN, Mustafa Can GÜLER, Erdem TOKTAY, Elif POLAT, Hatice Tuğçe GEDİK

**Affiliations:** 1 Department of Physiology, Faculty of Medicine, Atatürk University, Erzurum, Turkey; 2 Department of Physiology, Faculty of Medicine, Yüksek İhtisas University, Ankara, Turkey; 3 Department of Physiology, Faculty of Medicine, Yozgat Bozok University, Yozgat, Turkey; 4 Department of Histology and Embryology, Faculty of Medicine, Kafkas University, Kars, Turkey; 5 Department of Biochemistry, Faculty of Nutrition and Dietetics, Health Sciences University, Erzurum, Turkey; 6 Department of Anatomy, Faculty of Medicine, Atatürk University, Erzurum, Turkey

**Keywords:** Renal ischemia-reperfusion injury, inflammation, oxidative stress, *Tarantula cubensis* extract

## Abstract

**Background/aim:**

Renal ischemia-reperfusion (IR) related acute kidney injury (AKI) is an important health problem and has not yet been fully treated. *Tarantula cubensis* extract (TCE) is a homeopathic drug that has antiinflammatory and antioxidant effects. This study aimed to investigate the effects of TCE on renal ischemia-reperfusion injury in rats.

**Materials and methods:**

This study was carried out on 48 Spraque-Dawley male rats, which were divided into six groups. The first, second, and third groups were control, sham, and IR groups, respectively. Group four received IR and 0.2 mL of 96% ethanol. Group five and six received ischemia and reperfusion and TCE 0.01 and 0.1 mg per rat (which correspond to approximately 0.04 mg/kg, and 0.4 mg/kg), respectively. Tumor necrosis factor alpha (TNF-α), interleukin-1beta (IL-1β), total antioxidant status (TAS), and total oxidant status (TOS) levels in renal tissue were measured by enzyme-linked immunosorbent assay (ELISA). Oxidative stress index (OSI) was obtained by proportioning TAS and TOS. Superoxide dismutase (SOD), myeloperoxidase (MPO) activities, and malondialdehyde (MDA) levels were determined by manual spectrophotometric methods. The histopathological changes were evaluated via hematoxylin-eosin and immunohistochemical staining.

**Results:**

In IR group, renal tissue TNF-α and IL-1β levels were significantly higher than control group (p < 0.0001 for both), and low (p < 0.0001 for both) and high dose (p < 0.0001 for both) TCE administration decreased these markers. Low and high doses of TCE decreased OSI values compared with IR group (p = 0.04 and p = 0.001 respectively). Although TCE decreased MDA levels, it was not statistically significant. MPO levels significantly decreased. In addition, TCE has been found to prevent hemorrhage, cast formation, and dilatation caused by IR in renal tissues stained with hematoxylin-eosin. And also, the most intense nuclear factor kappa B (NFκB) and caspase-3 immunopositivity found in IR group was decreased in both of the TCE groups.

**Conclusion:**

Although TCE showed a protective effect by inhibiting inflammation against IR damage in renal tissues, there was no clear effect on oxidative stress. Larger and more detailed studies are needed to clarify the issue.

## 1. Introduction

Ischemia is insufficient perfusion of organs or tissues due to partial or complete interruption of blood flow. The reduction of oxidative phosphorylation with ischemia causes the depletion of energy stores, the inhibition of the sodium-potassium ATPase (Na-K ATPase) pump working with adenosine triphosphate (ATP) in the cell membrane, and this causes increases in Na^+^ and Ca^+2^ in the cell, swelled cells and activated cytotoxic enzymes. In addition, these cellular changes increase the release of proinflammatory cytokines and inactivate the antioxidant enzyme system [1]. Reperfusion is required for repairing damaged membranes and organelles caused by ischemia, and removing of toxic metabolites may further increase oxidative stress and inflammation, paradoxically causing further damage than the damage caused by ischemia [2–4].

Acute kidney injury (AKI) is a life-threatening clinical condition characterized by a decrease in urine output, the accumulation of waste material in the blood due to the inability to excrete molecules such as urea and creatinine, and that occurs with a sudden decrease in kidney functions [5].

AKI caused by renal IR, which develops in major cardiovascular operations, urological procedures, kidney transplantation, trauma, sepsis, or hypovolemia, is unfortunately still an important health problem [6]. Therefore, extensive studies have been conducted to find the prevention or treatment of IR-induced AKI, but no clear treatment has been found yet. In the development of AKI due to renal IR, the deterioration of the renal tubule structure, hemodynamic changes, increased oxidative stress due to the increase of oxidant substances and the inhibition of the antioxidant system, and the increase in inflammation with activation of proinflammatory cytokines and immune cells are responsible. Recent studies have also focused on treating or preventing these factors.

*Tarantula cubensis* is a spider member of the genus Mygale, consisting of large, mouse-shaped hairy tarantulas, and its venom can cause serious systemic reactions such as renal failure, intravascular coagulation, and thrombocytopenia [7]. *Tarantula cubensis* extract (TCE) was obtained by processing the whole spider according to the rules of “Pharmacopeia Germanica” and diluting it with alcohol.

In some studies, it has been found that TCE has regenerative, anticancer, antiinflammatory, and resorptive effects [8, 9]. TCE is effective in treatments of bluetongue in cattle [10], oral papillomatosis [11] and breast tumors in dogs [12], indolent ulcers in cats [13], thrombus in the artery [14], hand-foot-mouth disease [8] and endometriosis in cattle [9].

In a study of liver damage with aflatoxin in rats, it has been shown that TCE can reduce the negative effects of aflatoxin on the liver by activating the antioxidant system [15].

In another study on peripheral nerve damage, it was shown that TCE reduced caspase-3 and NF κB immune reactivity, reduced axonal and myelin damage, and could have neuroprotective activity [16].

In our previous study, in the sepsis model induced by the cecal ligation puncture, TCE significantly reduced TNF-α, IL-1β, IL-6, TOS, OSI levels, increased TAS values, and significantly decreased caspase-3 and NF κB expression. Thus, it was concluded that TCE reduces lung damage in polymicrobial sepsis with its antiapoptotic, antioxidant and antiinflammatory activities [17].

The aim of this study was to investigate the effects of TCE on oxidative stress, apoptosis, and inflammation in renal IR injury by evaluating proinflammatory markers TNF-α, IL-1β, IL-6 cytokines, NF-κB immune positivity, oxidative stress status with TAS, TOS, MDA, SOD, and MPO levels. We evaluate the protective effects of TCE in renal tissue with immunohistochemical changes.

## 2. Materials and methods

### 2.1. Animal research ethics

In this study, 48 Spraque-Dawley male rats (250–300 g) were obtained from Atatürk University’s Experimental Animal Laboratory at the Medicinal and Experimental Application and Research Center. The animal experiments and procedures were approved by Atatürk University Animal Experiments Local Ethics Committee (2017, No:70). It was calculated with the G power program that 7 animals should be present in each group for significance at 80% power and 95% confidence level. Considering the losses that may occur, a total of 48 animals, 8 animals in each group, were included in the study.

Every two rats were housed in standard plastic cages with sawdust bedding at stable temperature (22 ± 3 °C), humidity (55 ± 10%) with a 12 h light/dark cycle by Atatürk University’s Experimental Animal Laboratory’s trained staff.

All animals were placed under the above conditions and acclimatized to the facilities with standard rat food and tap water ad libitum for one week, then fasted for 12 h with water ad libitum before the experiments. The rats were randomly divided into 6 groups. Blind analysis was provided by not giving information about the groups of samples given to the researchers, who made biochemical and histopathological examinations. All of the procedures and analyzes were carried out at the same time.

### 2.2. Experimental design

In this study ketamine HCl (Ketalar, Eczacibasi, Turkey) (intramuscularly 100 mg/kg) and xylazine hydrochloride (Rompun, Bayer, Germany), (20 mg/kg) were given for general anesthesia and then surgical procedures were applied. For the IR procedure, after shaving the abdominal areas of the rats and providing local sterilization with 10% polyvinylpyrrolidone iodine (Batticon, Adeka), a dorsal retroperitoneal 3 cm incision was made. Right nephrectomy was performed by dissecting the right renal pedicle and ligating the artery, vein, and ureter. Left kidney arteria and veins were held with an atraumatic microvascular clamp for 1 h. At the end of one hour, the clamp was opened, and the kidney was placed back in the retroperitoneal area and closed with 3/0 polyglactin (Vicryl). The rats that were placed in their cages were kept for 24 h for reperfusion. At the end of 24 h, the left kidneys of the rats were taken and sacrificed under general anesthesia.

Group 1 (control): No application was performed, kidney tissues were removed and rats were sacrificed.

Group 2 (sham): Dorsal retroperitoneal incision was made in rats under general anesthesia. It was sealed with 3/0 polyglactin (Vicryl, Ethicon) without any further action.

Group 3 (IR): Right kidney nephrectomy was performed, and 24 h reperfusion was applied to the left kidney after 1 h of ischemia.

Group 4 (IR+vehicle (alcohol): IRA): The rats were subcutaneously administered 0.2 mL of 96% ethanol 30 min before the surgical procedure. After 30 min, the IR procedure was carried out.

Group 5 (IR+low dose TCE: IRL): TCE was administered subcutaneously at a dose of 0.01 mg 30 min before the IR procedure.

Group 6 (IR+high dose TCE: IRH): TCE was administered subcutaneously at a dose of 0.1 mg 30 min before the IR procedure.

A total of 48 animals were included in the experiment. Six of them were lost for various reasons. The final number of animals in each group can be seen in Table 1.

### 2.3. Preparation and application of Tarantula cubensis extract

Tarantula cubensis extract (Theranekron_D6) was obtained from Richter Pharma AG Wels, Austria. Since TCE was obtained by dissolving in alcohol, in IRA group 0.2 mL of 96% ethanol, 0.01 mg (approximately 0.04 mg/kg) TCE in IRL group, and 0.1 mg (approximately 0.4 mg/kg) TCE in IRH group were administered subcutaneously 30 min before the IR protocol.

### 2.4. Collection and storage of tissue samples

Rats were sacrificed with an overdose of general anesthetic (thiopental sodium, 50 mg/kg). The rats were dissected on a frozen glass and samples were taken. Based on the data from this study, the potential benefits outweigh the deleterious effects on live rats that were minimized.

Some pieces of the kidney were taken and preserved in 10% formaldehyde solution for histopathological examination. The remaining parts of the kidney, which were buffered and homogenized, were centrifuged at 2800 g for 20 min and taken into microcentrifuge tubes (Eppendorf AG, Germany) and stored at −80 °C for ELISA and manual biochemical tests.

### 2.5. Sample collection and preparations of analysis

Tissue supernatants stored at −80 °C until the day of the experiment were dissolved by keeping them at −20 °C and +4 °C, respectively. Clear supernatants formed by centrifugation at +4 °C 2800 g for 10 min were used.

### 2.6. Assessment of oxidative stress parameters

The oxidative stress index is calculated as the total oxidant level (TOL)/total antioxidant level (TAL) × 10. Unit is Trolox equivalent/L.

### 2.7. Measurement of biochemical parameters in kidney

One hundred milligrams of kidney tissue samples were homogenized with 2 mL phosphate buffer and centrifuged at 2500 g, at +4 °C for 20 min, and supernatants were placed in 2 mL Eppendorf tubes and stored at −80 °C until the analysis date.

MDA (micromol/mg protein), MPO (U/mg protein), and SOD (SOD U/mg protein) analyzes were measured manually. TNF-α (pg/mg) (E-EL-R0019-Elabscience-China), IL-1β (pg/mg) (E-EL-R0012, Elabscience, China) were measured with enzyme-linked immunosorbent assay kits in ELISA reader (ELISA, BioTEK Powerwave XS Winooski, United Kingdom) [18, 19].

Serum TOS was measured using Erel’s TOS method (Rel Assay Diagnostics, Turkey). The assay is based on the oxidation of ferrous ions to ferric ions in the presence of various oxidant species in an acidic medium. The ferric ion makes a colored complex with xylenol orange. The color intensity is related to the total amount of oxidant molecules present in the sample [18]. The results were expressed as micromol/mg protein.

Serum TAS was quantified by a colorimetric measurement method developed by Erel. In this method, the antioxidant in the sample, the dark blue-green colored 2,2′-azino-bis (3-ethylbenzothiazoline-6-sulfonic acid) (ABTS), is a radical against the colorless reduced ABTS form. A change in absorbance at 660 nm is related to the total antioxidant level in the sample. The results were expressed as mmol/mg protein [20].

### 2.8. Statistical analysis

Data were evaluated using SPSS statistical software package 25.0 for Windows

((IBM Corporation, Armonk, NY, USA), biochemical data were analyzed by ANOVA with posthoc LCD test. And Pearson test was performed for correlation analysis. In the histopathological examination, the difference between the groups of data obtained semiquantitatively was determined by the Kruskal-Wallis test, and the determination of the groups that made the difference was determined by the posthoc Dunn-Bonferroni test. A p < 0.05 value was considered statistically significant. All statistical significance was reported at p ≤ 0.05.

### 2.9. Histopathological examination

Serial 5 μm sections were taken from the paraffin blocks to contain the same cell with the microtome (Leica RM2145). Five-micron sections were taken from the whole tissue block onto positively charged slides. Slides were kept under suitable conditions for hematoxylin and eosin staining and immunohistochemical (IHC) staining. At the end of the deparaffinization process, 5-micron sections were taken on a positively charged slide, and histochemical staining was performed with caspase-3 (Santa Cruz Bio., China) and nuclear factor kappa B (NF-κB) (Santa Cruz Bio., China) antibody in Ventana Benchmark GX automatic immunohistochemistry stainer.

Light and IHC Staining Preparations Olympus BH 40 brand light microscope with camera attachment was photographed and interpreted. The sections examined under the light microscope were scored as none (−), mild (+), moderate (++), and severe (+++).

## 3. Results

A significant difference was found among the groups in terms of TNF-α. The highest TNF-α value was found in IR group and the lowest value in IRH group.

**Figure 1:**
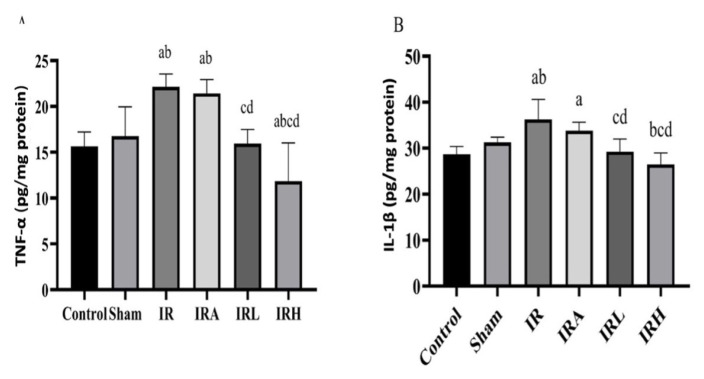
Comparison of TNF-α and IL-1β values among groups. Inflammatory markers in the kidney. A) TNF-α levels and B) IL-1β levels. Data are expressed as mean ± SD (n = 8). a: p < 0.05 versus to control group, b: p < 0.05 versus to sham groups. c: p < 0.05 versus to IR group, d: p < 0.05 versus to IRA group.

In posthoc analysis in IR group, TNF-α levels were significantly higher than the levels in sham, control, IRL, and IRH groups (p < 0.001 for all groups). There was no significant difference between IR group and IRA. In addition, TNF-α levels in IRA group were significantly higher than the levels of sham (p < 0.01), control (p < 0.001), IRL (p < 0.01), and IRH (p < 0.001) groups. In IRH group, TNF-α levels were lower than the levels of control group (p < 0.01). TNF-α levels were higher in IRL group than the levels of IRH group (<0.01). There was no statistically significant difference between sham and control groups (Figure 1a).

Renal tissue IL-1β levels were lower in sham (p < 0.01), control (p < 0.001), IRL (p < 0.001), and IRH (p < 0.001) groups compared to IR group. IL-1β levels were similar in IR and IRA groups. There was no difference in IL-1β levels among control, IRL and IRH groups. IL-1β levels were significantly lower in IRH group than the levels of IRL group (p < 0.05). In IRA group, IL-1β levels were significantly higher than control (p < 0.01), IRL (p = 0.01), and IRH groups (p < 0.001) (Figure 1b).

**Figure 2:**
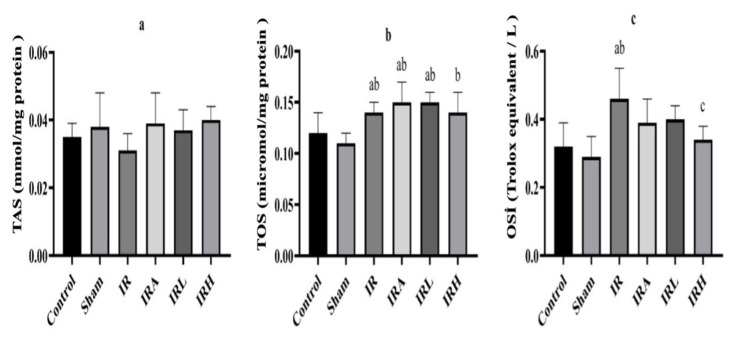
Comparison of TAS, TOS, and OSI levels in groups. TAS (mmol/L), TOS (μmol/L) and OSI values results are presented as mean ± SD (n = 8). a: p < 0.05 versus to control group, b: p < 0.05 versus to sham groups. c: p < 0.05 versus to IR group, d: p < 0.05 versus to IRA group.

There was no significant difference among the groups in terms of renal TAS levels (Figure 2a). A significant difference was found among the groups in terms of renal tissue TOS levels (Figure 2). Renal TOS levels in the IR and IRA groups were higher than the levels of control and sham groups (p < 0.01). Mean TOS levels in IRL and IRH groups were similar to those in IR group. In addition, there was no statistically significant difference between IR and IRA groups (Figure 2b).

Significant difference was found among all of the groups in terms of renal tissue OSI values.

OSI values in IR group were significantly higher than the levels in sham and control groups (p < 0.001 for both). In IRL and IRH groups, OSI values were lower than the levels in IR group (p < 0.005 and p < 0.01, respectively). No significant difference was detected between IR and IRA groups (Figure 2c).

**Figure 3:**
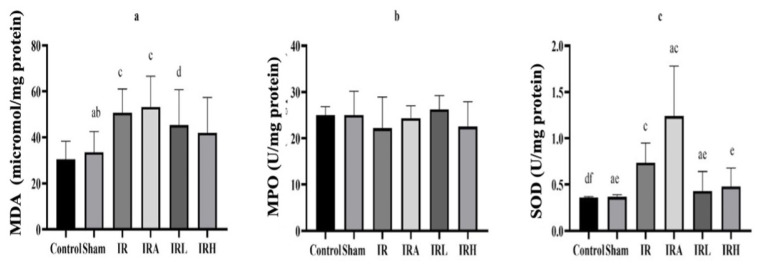
Comparison of MDA, SOD and MPO levels in groups. Data are expressed as mean ± SD. a: p < 0.05 versus to control group, b: p < 0.05 versus to sham groups. c: p < 0.05 versus to IR group, d: p < 0.05 versus to IRA group.

A significant difference was found between the groups in terms of renal tissue MDA levels (Figure 3).

Mean renal MDA levels were significantly higher in IR and IRA groups than in control (p < 0.01 for both) and sham groups (p < 0.05 for both). MDA levels were found to be lower in IRL and IRH groups than the levels in IR and IRA groups, but this decrease was not statistically significant (Figure 3a).

Data are expressed as mean ± SD. a: p < 0.05 versus to control group, b: p < 0.05 versus to sham groups. c: p < 0.05 versus to IR group, d: p < 0.05 versus to IRA group.

There were significant differences among the groups in terms of renal tissue MPO levels (Figure 3). Renal tissue MPO activity in IRA group was significantly higher than control, sham, IRL, and IRH groups (p < 0.001 for all). In addition, MPO activity in IRA group was higher than IR group (p < 0.01). The mean MPO activity of IR group was statistically higher than those of control (p < 0.001), sham (p < 0.05), and IRL groups (p < 0.05). MPO activity results were similar in IRL, and IRH groups (Figure 3b).

There was no significant difference among all groups in terms of average SOD values (Figure 3c).

### 3.1. Histopathological findings

**Figure 4:**
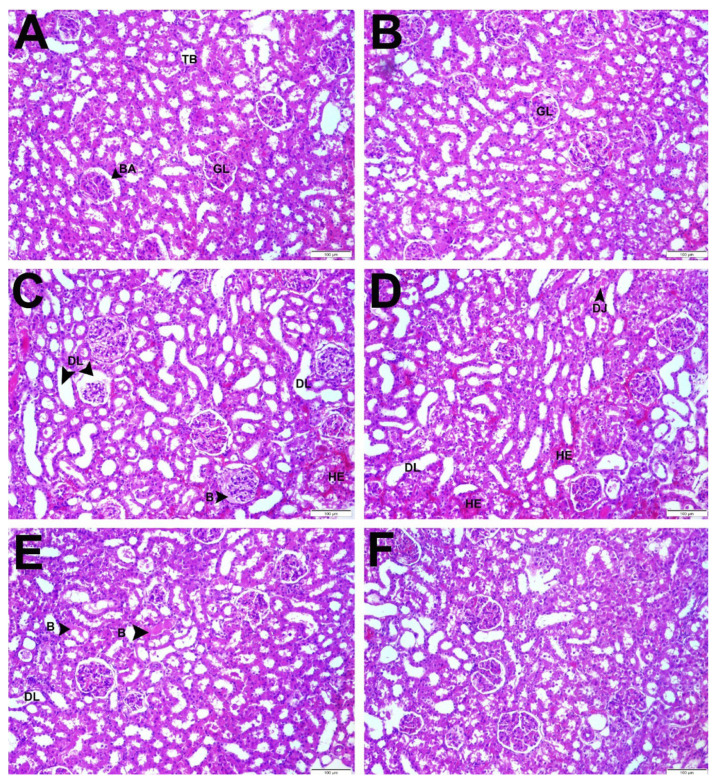
Demonstration of changes in kidney tissue with hematoxylin and eosin. **Star:** cast formations, **triangle:** degenerative tubule, **round ring: p**ositive cells, **arrow:** hemorrhage.

The histopathological effects of low and high dose TCE administration to rats 30 min before the IR procedure against renal IR injury are shown in Figure 4.

Control group: It was observed that the glomerular structures in the renal cortex were normal and the distal and proximal tubules were healthy (Figure 4a).

Sham group: Similar to control group, healthy kidney tissue was observed (Figure 4b).

IR group: While significant dilatations and accumulations were observed in some glomerular spaces in the renal cortex, significant degeneration and cast formation were observed in the distal and proximal tubules. In addition, hemorrhage was detected in the interstitial space between the tubules (Figure 4c).

IRA group: In addition to similar findings to IR group, there were prominent degeneration and cast formation in tubular structures. Also, when compared to IR, more widespread hemorrhage was observed in the interstitial area between the tubules in this group (Figure 4d).

IRL group: While mild dilatations were seen in some glomeruli, degenerations were observed in tubule structures. However, hemorrhage was not observed in this group (Figure 4e).

IRH group: Findings similar to control group were dominant in this group. Glomeruli and glomerular spaces were normal in size and shape. Renal tubules were in healthy appearance (Figure 4f).

**Figure 5:**
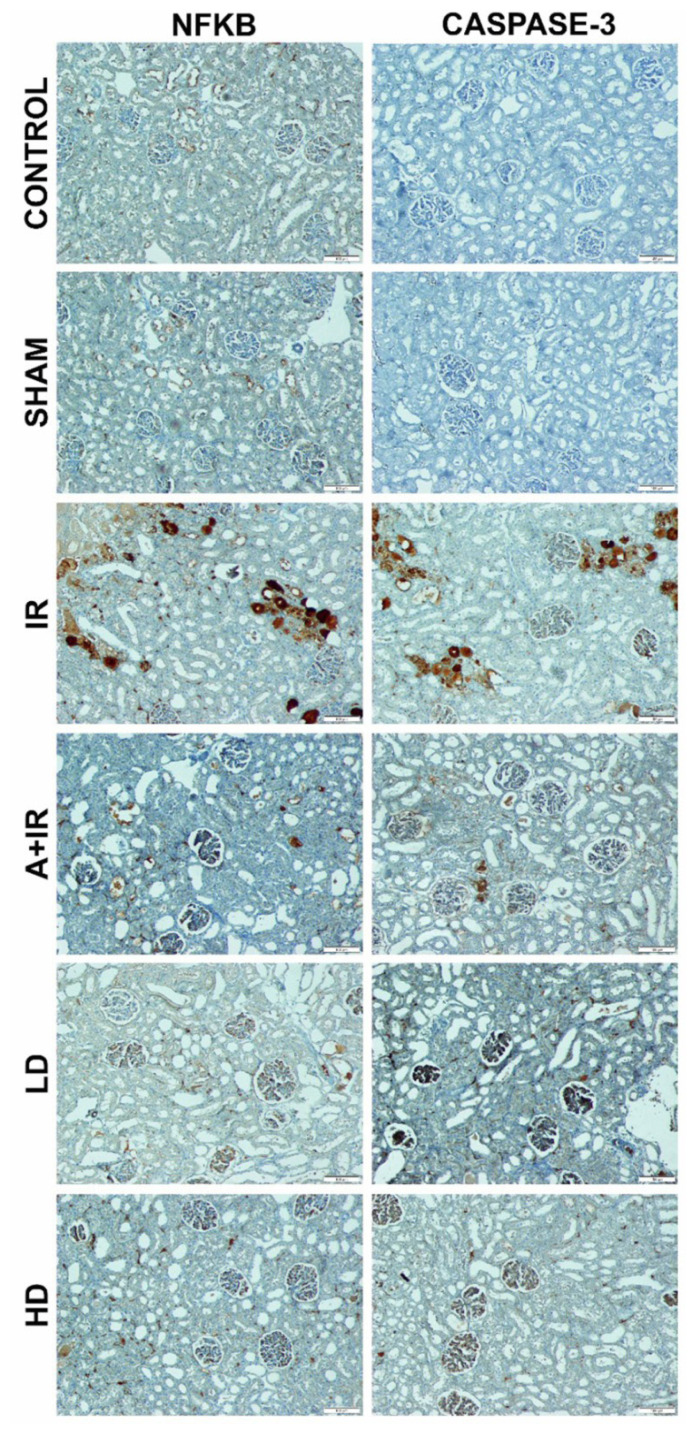
IHC evaluation with NF-κB and caspase-3.

Histopathological damage based on the presence of tubule degeneration and hemorrhage was scored as − (none), + (little damage), ++ (moderate damage), +++ (severe damage) (Table 1). All these values were collected in each group and shown graphically. IHC evaluation with NF-κB and caspase-3 was shown in Figure 5.

**Figure 6:**
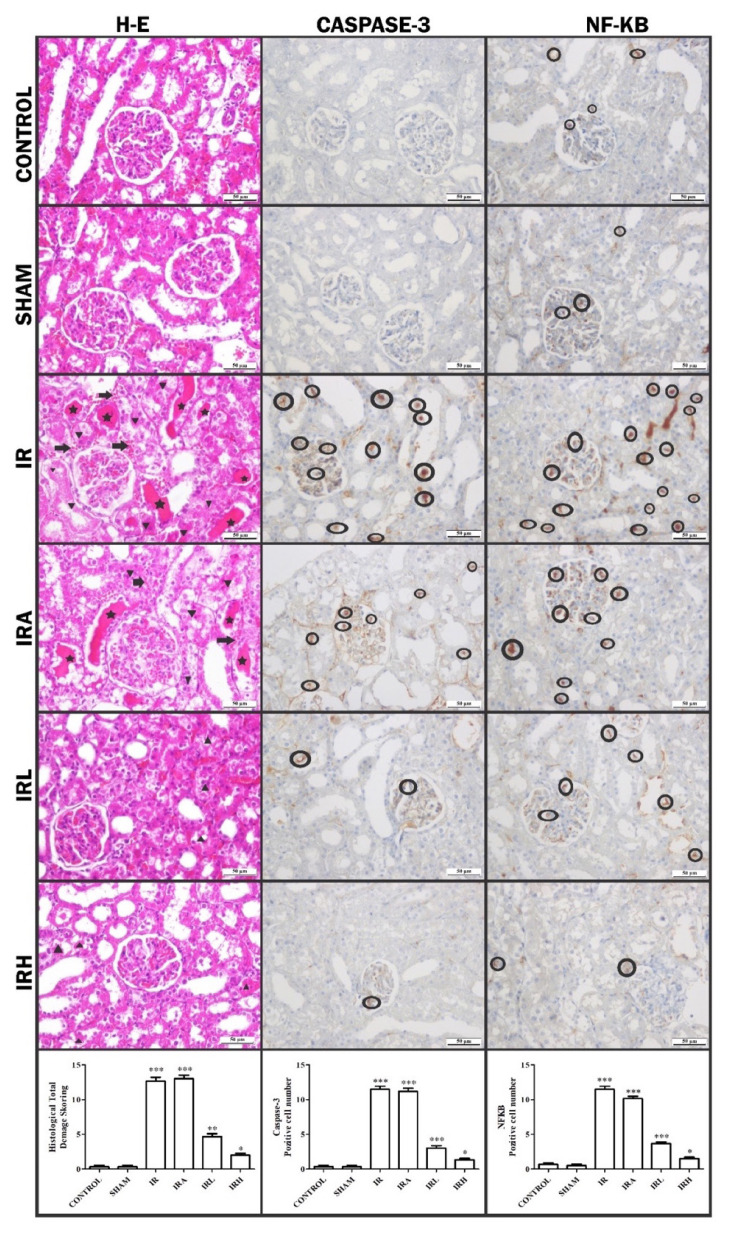
The statistically evaluation of positive cells and histological total damage scoring in each group.

In IHC evaluation, NF-κB and caspase-3 immune positivity were scored as − (none), + (low), ++ (moderate), +++ (severe). While moderate immune positivity was observed in IRA group and severe immune positivity in IR group, mild immune positivity of NF-κB was observed in control, sham, IRL, and IRH groups (Table 2). Positive cells in different areas in each group were counted and statistically evaluated (Figure 6).

The statistically evaluation of positive cells and histological total damage scoring in each group

While IHC staining with caspase-3 antibody showed mild immunological positivity in IRH group, moderate in IRA and IRL groups and severe in IR group, immune negativity was observed in control and sham groups (Table 2).

## 4. Discussion

In our study, possible protective effects of TCE, which is a homeopathic drug, in IR damage were investigated for the first time. It was determined that TNF-α and IL-1β, which are inflammatory cytokines, increased in IR group, and TCE decreased these two markers in both low and high doses. Although there was no significant difference among the groups in terms of TAS values indicating the total antioxidant level, oxidative stress markers TOS, OSI and MDA were higher in IR group. TCE administration caused a decrease in OSI levels in dose-dependent manner.

While tissue MPO activity increased in IR group, TCE decreased it. Histopathological examination showed that both TCE doses decreased apoptosis and renal tissue damage, such as decreasing tubules degeneration, hemorrhage, and cast formation. And high dose of TCE was more effective than the low dose of TCE administration. In our literature review, no study similar to the histopathological data of TCE was found. To the best of our knowledge, this study is the first on this subject.

Models used for the development of kidney IR injury vary among species. The most commonly described one is the temporary closure of only renal artery or renal artery and vein together. Studies have shown that clamping renal arteries and veins together is the ideal method for consistent maximal damage [21, 22].

The ischemia period required to achieve irreversible kidney damage is typically longer in larger animal models such as dogs and pigs than the rodent models [23]. This marked difference in IR tolerance makes it difficult to apply similar designs among species. Prolonged ischemia is associated with more consistent damage than shorter durations; therefore, during model development, researchers often want the maximum tolerable ischemia time for animals used [24]. There are many studies in which IR procedure times differ in rats. There are many studies where different IR times have been applied. In this study, 1-h ischemia and 24-h reperfusion periods were applied, as used in studies investigating oxidative stress and inflammation [25, 26].

In our study, unilateral IR was applied, while contralateral nephrectomy protocol was used [22, 27]. Also, we performed retroperitoneal incision because it was less traumatic [24].

In our study, TCE did not affect SOD activity. TCE did not increase TAS values, either. With this result, it can be said that TCE shows its effect without causing any change in antioxidant levels in the renal IR model.

In a study conducted in rats, the effects of TCE on the toxic effects of aflatoxin on the liver and other organs were investigated [15]. In the aflatoxin group, it was observed that MDA levels increased in kidney tissue and MDA levels decreased with TCE administration. In the same study, it was determined that renal SOD activity decreased in the group given TCE alone. While aflatoxin caused an increase in SOD, administration of TCE following aflatoxin caused a significant decrease in SOD. At the same time, administration of TCE alone or with aflatoxin has been found to reduce the activities of other antioxidant enzymes CAT and GSH-Px. Considering these results, it was concluded that TCE may have an antioxidant property alone and thus prevent the oxidant effect of aflatoxin, and therefore activation of other defense systems may not be required [15]. But in our study, TCE was not able to reduce MDA. In addition, TCE did not cause a significant change in SOD activity in our study. This may be due to the different treatment protocols of TCE and experimental models that cause oxidative stress.

In a study performed on cattle with hand-foot-mouth disease, Lotfollahzadeh et al. found that subcutaneous TCE caused a decrease in oral mucosal lesions [8]. Also, TCE was found to reduce endometriotic foci and histopathological scores in rats with experimental endometriosis [9]. Additionally, it was found that TCE increased epithelization in wound healing and accelerated the production of granulation tissue [28].

In one study conducted on rabbits, experimental tendon damage was created and the effect of TCE was examined. In this study, TCE decreased the cells related to inflammation [29]. Gultiken et al. investigated the effects of TCE on the breast tumor of dogs. TCE has been shown to reduce Ki-67, showing mitotic activity, and Bcl-2, inhibiting apoptosis [30]. In our study, TCE reduced caspase-3, which is an apoptosis biomarker, in the histopathological examination of renal tissues. This result is different from the results of the study mentioned above. While apoptosis is necessary for the treatment of breast tumors, it is an unwanted situation for IR, because of causing increased damage in IR. In our study, inhibition of apoptosis by TCE contributed to the reduction of IR-induced renal tissue damage. These different effects of TCE can depend on the type of pathology, the tissue, and the dose given. In another study conducted in cattle, it was observed that TCE decreased papillomatosis [31].

In addition, in another study, we created sepsis by cecal ligation puncture method in rats, TCE significantly reduced TNF-α, IL-1β, IL-6, TOS, OSI levels, expression of caspase-3 and NF κB and increased TAS values. The number of *Escherichia coli* colonies decreased in the lung, heart, liver and kidney tissues of the group treated with TCE. We concluded that TCE reduces lung damage in polymicrobial sepsis with its antiapoptotic, antioxidant, and antiinflammatory activities [17]. Dik et al. showed that the levels of TGF-β, VEGF, AFP, and caspase-3 levels in rats with colon cancer were decreased by TCE [32]. A study with human cancer cells suggested that TCE was selectively toxic for cancer cells via inducing caspase-3 mediated apoptosis [33]. Er et al. found that TCE administration reduced tumor mass score in rats with colon cancer. TCE increased TNF α levels but it was not statistically significant, although the present study suggested that TNF-α levels were decreased with TCE administration. There was no significant changes in MDA levels with TCE administration like in the present study [34]. In our study, the effects of TCE on renal IR were examined and observed that histopathological findings were improved.

TCE administration decreased renal tissue levels of proinflammatory cytokines such as TNF-α and IL-1β. This data suggested that TCE had alleviated inflammation. It has also been determined that TCE reduces oxidative stress and inflammation due to IR. The protective effects of TCE on tubules degeneration were higher in 0.1 mg dose than in 0.01 mg. The decrease of TNF-α and caspase-3 levels were higher in high dose TCE than in low dose. As a result, high dose of TCE might be more effective in reducing tubules degeneration, inflammation and apoptosis. More detailed studies with different doses are needed to confirm this.

## 5. Conclusion

In conclusion, TCE was found to reduce IR damage, proinflammatory markers, oxidative stress index especially at high doses, and histopathological examination showed that accumulation in glomerular spaces, dilatation in glomeruli and tubules, and hemorrhages in the intertubular interstitial area were decreased with TCE. In IHC staining, TCE was found to reduce apoptosis and inflammation caused by IR.

According to these findings, it can be said that TCE reduces IR damage. To verify these results, further experimental studies that examine enzymatic-nonenzymatic antioxidants and other oxidative stress markers at molecular level are needed.

## Figures and Tables

**Table 1:** Table of histopathology staging.

Groups	Degenerative tubules	Hemorrhage
Control (n = 8)	**− (** * * **)**	**−** (**)
Sham (n = 6)	**− (** * * **)**	**−** (**)
IR (n = 7)	**++**	**+**
IRA (n = 5)	**− (** * * **)**	**++ (** * **)**
IRL (n = 8)	**+ (** * **)**	**−** (**)
IRH (n = 8)	**− (** * * **)**	**−** (**)

(Histopathological results were analyzed by Kruskal-Wallis and posthoc Dunn-Bonferroni tests.

*Compared with IR *; p < 0.05, **; p < 0.01)

**Table 2:** Immunohistochemical scoring.

Groups	NF-κB	Caspase-3
Control	+ (**)	− (***)
Sham	+ (**)	− (***)
IR	**+++**	**+++**
IRA	++(*)	++ (*)
IRL	+ (**)	++ (*)
IRH	+ (**)	+ (**)

(Immunohistochemical results were analyzed by Kruskal-Wallis and posthoc Dunn-Bonferroni tests. * Compared with IR *; p < 0.05, **; p < 0.01, ***; p < 0.001)
